# An In Vitro Investigation of Levofloxacin-Induced Cytotoxicity in Rat Bone Marrow Mesenchymal Stem Cells

**DOI:** 10.7759/cureus.77802

**Published:** 2025-01-21

**Authors:** Abdul Rehman Arif, Jiadong Yu, Qingshan Yin, Yu Deng

**Affiliations:** 1 Department of Orthopedic Trauma and Orthopedics, Zhongnan Hospital of Wuhan University, Wuhan, CHN; 2 Department of Orthopedic Trauma and Microsurgery, Zhongnan Hospital of Wuhan University, Wuhan, CHN; 3 Department of Orthopedics, Tianmen First People's Hospital, Tianmen, CHN

**Keywords:** apoptosis, bone marrow mesenchymal stem cells, cytotoxicity, fluoroquinolone, levofloxacin

## Abstract

Background: Levofloxacin, a widely used fluoroquinolone antibiotic, has been linked to musculoskeletal complications. However, its impact on bone marrow mesenchymal stem cells (BMSCs), which are vital for tissue repair and regeneration, remains poorly understood.

Aim: This study aims to examine the impact on rat BMSCs following therapy with levofloxacin.

Methods: Rat BMSCs were exposed to various doses of levofloxacin (0, 14, 28, 56, 112, and 224 μM) to assess its possible cytotoxic impact on these stem cells. Cell viability was assessed using the MTT assay to evaluate the cytotoxic effects of levofloxacin. Cell apoptosis was calculated with Annexin V-fluorescein isothiocyanate (FITC)/propidium iodide (PI) double staining, along with the expression levels of matrix metalloproteinase-3 (MMP-3), MMP-13, collagen type I alpha 1 (Col1A1), tissue inhibitor of metalloproteinase-1 (TIMP-1), and TIMP-3 messenger RNA (mRNA), which were assessed using RT-PCR. An apoptotic marker, caspase-3, was detected by immunocytochemical analysis.

Results: In certain concentrations (0-224 μM), as the concentration of levofloxacin increased, the number of apoptotic cells increased. The results demonstrated that levofloxacin significantly upregulated the mRNA levels of MMP-3 as well as MMP-13 in a dose-related manner, simultaneously downregulating TIMP-1 expression. In contrast, the expression of TIMP-3 and Col1A1 remained unaffected. In addition, the expression of caspase-3 was substantially elevated by levofloxacin in a concentration-related manner, between 28 μM and 224 μM, as indicated by immunocytochemistry.

Conclusion: These findings provide evidence that levofloxacin exerts cytotoxicity on BMSCs, shown by increased apoptosis and a reduction in extracellular matrix components, highlighting a potential adverse impact of levofloxacin. Additionally, this cytotoxic effect may negatively affect fracture healing and impair the regenerative capacity of BMSCs.

## Introduction

Quinolones, especially third- and fourth-generation quinolone drugs, have a wide antimicrobial spectrum and strong antibacterial activity [1]. Quinolones are frequently utilized as antimicrobial agents to prevent infection and management in orthopedic fracture surgeries. But at the same time, quinolones have various adverse reactions [2,3]. Recent studies have shown that quinolone drugs have adverse effects on the skeletal muscle system, including Achilles tendon rupture, muscle pain, and joint pain [4,5]. Past research has demonstrated that quinolone drugs exert toxic effects on tendon cells, leading to apoptosis. Specific alterations include decreased expression of certain extracellular matrix (ECM) proteins, inhibited tendon cell proliferation, increased expression of matrix metalloproteinases (MMPs), and induced ultrastructural degeneration [4,6]. Similar toxic effects have been observed in anterior cruciate ligament cells, synovial cells, and cartilage cells [7-9]. Research has established that alterations in ECM component synthesis and degradation play a crucial role in fluoroquinolone-induced apoptosis [7]. Studies have shown that fluoroquinolones, specifically levofloxacin and ciprofloxacin, have been shown to induce a time- and concentration-dependent increase in apoptosis, mediated by the caspase-3 pathway in cultured human tenocytes [6,10]. Cell apoptosis must be considered a critical event in the process by which quinolone drugs inhibit cell proliferation.

Bone marrow mesenchymal stem cells (BMSCs) possess multipotent differentiation capabilities, enabling them to differentiate into various cell types of mesodermal origin under specific conditions. During fracture healing, BMSCs differentiate into osteoblasts, contributing to the regeneration and repair of bone tissue [11]. BMSCs can also be used as seed cells for repairing health organizations, knee joint cartilage, and meniscus [12]. While the cytotoxic effects of quinolones on tendon cells, synovial cells, cartilage cells, and osteoblasts have been well documented, comprehensive studies investigating their impact on BMSCs remain limited. Therefore, this experiment mainly studied the influence of the quinolone drug levofloxacin on BMSCs.

Levofloxacin is a kind of typical quinolone, has a good antibacterial effect and penetrability, and is widely used in clinical settings. However, it is worth noting that levofloxacin in clinical use may cause fracture non-unions, rupture of tendons, and a series of adverse reactions [4,5,13].

The outcome of levofloxacin on BMSCs, whether in vivo or in vitro, remains unclear. This investigation focused on elucidating levofloxacin-triggered cytotoxicity in BMSCs, focusing on the importance of the role of matrix-degrading enzymes along with the induction of cellular death. This research examined cell death, type I collagen formation, inhibitors of tissue of metalloproteinases (TIMPs), expression of MMPs, and the apoptosis marker caspase-3 in rat bone marrow stem cells treated with levofloxacin.

## Materials and methods

Isolation and cultivation of BMSCs

SD rats (100-120g) were sourced from the Laboratory Animal Center at Wuhan University (430071, China). Under ether anesthesia, the femur and tibia were removed from two legs under aseptic conditions. The bones were suspended in 10 ml of Dulbecco's Modified Eagle Medium (DMEM), which contained penicillin (100 U/ml), fetal bovine serum (FBS), and streptomycin (100 μg/ml), and biting open at both ends of the bones and rinsing out the bone marrow with a syringe. Cells were cultured at 37°C in a 5% CO₂ environment, and the medium was replaced every 96 hours. Once the cells reached confluency, they were trypsinized using a solution comprising 0.2% ethylenediaminetetraacetic acid (EDTA) along with 0.1% trypsin. Third-generation BMSCs were used for the investigations.

MTT assay

BMSCs were cultured in 96-well plates at a density of 1×10⁴ cells per well and treated with levofloxacin at concentrations of 0, 14, 28, 56, 112, and 224 μM for 24 or 48 hours. Cell viability was assessed using the MTT assay. After the treatment period, cells were incubated with MTT (50 μg/mL) at 37°C for four hours. The resulting formazan crystals were solubilized in dimethyl sulfoxide, and the optical density (OD) was measured at 570 nm using a microplate reader.

Annexin V-fluorescein isothiocyanate (FITC)/propidium iodide (PI) assay

Apoptotic cells were examined through double-staining via propidium iodide (PI) along with Annexin V-FITC. Cells were handled for 48 hours, washed with PBS, collected via trypsinization, centrifuged, and re-suspended into affinity buffer. A volume of 5 μL of Annexin V-FITC along with PI was added to the suspensions, and then they were incubated at the ordinary laboratory temperature for a duration of 15 minutes in the absence of light.

Reverse transcriptase polymerase chain reaction (RT-PCR) assessment of messenger RNA (mRNA) expression

Total RNA was isolated with the aid of TRIzol reagent (Takara, Dalian, China), as per the manufacturer’s protocols. RT-PCR was conducted for amplifying target genes (Table 1) using specific primers. The PCR amplification commenced with an initial heated start at 95°C for five minutes, followed by 40 heat cycles. Each cycle included DNA denaturation (94°C, 30 seconds), primer annealing (55-58°C, 30 seconds), and extension (72°C, 45 seconds). The reaction culminated in a final elongation phase at 72°C for seven minutes. The PCR resultants were isolated on a 1% agarose gel and observed using ethidium bromide staining. β-actin functioned as a reference for cDNA integrity.

**Table 1:** Primer sequence and corresponding PCR conditions Col1A1: collagen type І A 1; PCR: polymerase chain reaction; bp: base pairs; TIMP: tissue inhibitors of metalloproteinases; MMP: matrix metalloproteinase

Primer	Sequence	bp	Annealing temperature	Cycle number
β-Actin	F: ACGATGGAGGGGCCGGACTCATC R: AAAGACCTCTATGCCAACACAGT	314	56	20
Col1A1	F: AGAGCATGACCGATGGATTC R; TTGAGGTTGCCAGTCTGTTG	172	55	25
MMP-3	F: GCTCATCCTACCCATTGCAT R; GCTTGTGCATCAGCTCCATA	219	65	25
MMP-13	F: CCCTTGATGCCATTACTA R; GAAATCCCAGGTCAGATA	261	65	25
TIMP-1	F: TCCCCAGAAATCATCGAGAC R; TCAGATTATGCCAGGGAACC	250	55	20
TIMP-3	F: TGTGCAACTTTGTGGAGAGG R; GTACCCGAAATTGGAGAGCA	173	60	25

Immunocytochemical assessment

An immunocytochemical evaluation was conducted to assess caspase-3 protein levels in BMSCs exposed to levofloxacin. Subsequent to 48 hours of treatment, cells were fixated using paraformaldehyde and treated with a rat anti-active caspase-3 polyclonal antibody, followed by a goat anti-rabbit IgG secondary antibody. DAB was incorporated as a chromogen, and visuals were collected through an Olympus BX60 microscope (Olympus Corporation, Japan). The intensity of staining was quantified utilizing Image Pro Plus software (version 6.1) and denoted as integrated optical density (IOD).

Statistical analyses

For quantitative results, each experiment was done three times separately. The data were presented as mean ± standard deviation. ANOVA and post hoc Dunnett-t-test were used to compare groups using SPSS Statistics for Windows, Version 13 (Released 2005; SPSS Inc., Chicago, United States). A p-value less than 0.05 was deemed statistically significant.

Ethical statement

All procedures in this study were reviewed, approved, and conducted in strict accordance with the ethical guidelines established by the Ethical and Research Committee of the School of Medicine, Wuhan University, China. The study adhered to internationally accepted ethical standards to ensure the welfare, humane treatment, and proper care of the animals used in the experiments.

## Results

MTT assay

**Figure 1:**
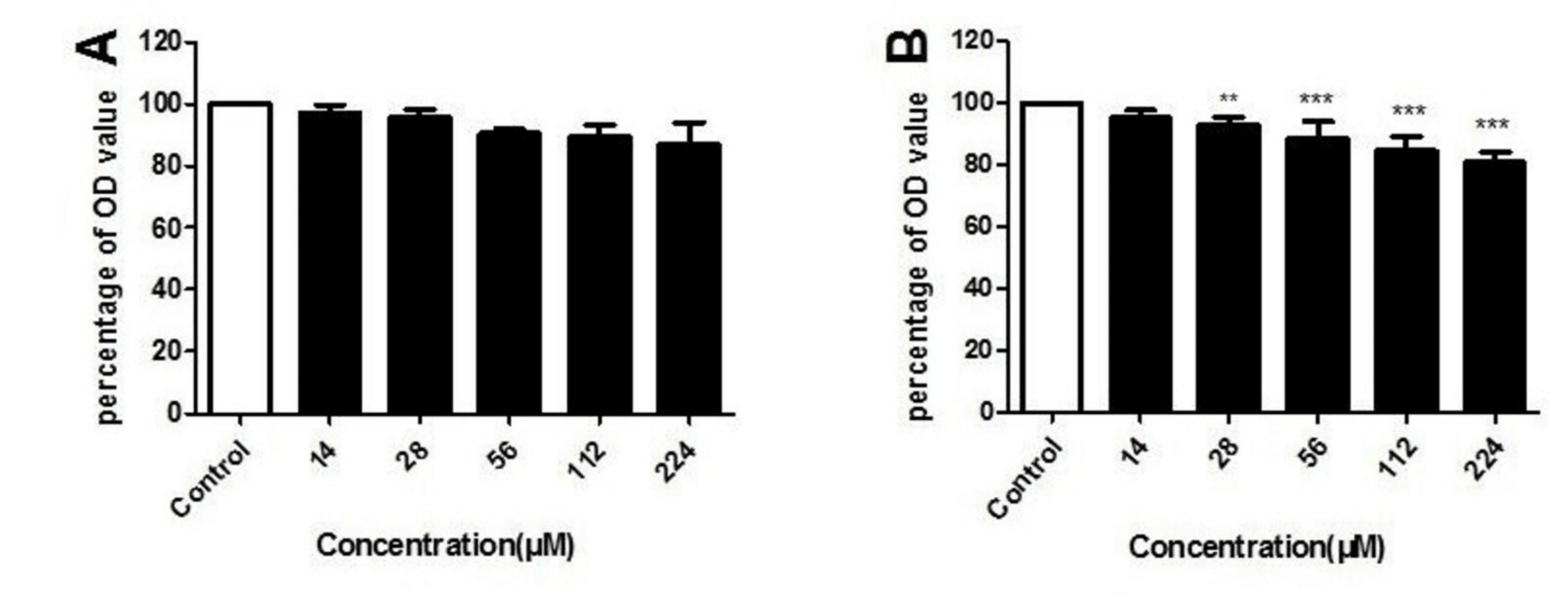
BMSC viability using MTT assay following levofloxacin exposure A: BMSCs were exposed to levofloxacin (concentration range: 14-224 μM) over a 24-hour period; B: BMSCs were exposed to 14-224 μM levofloxacin for 48 hours. Cell survival rates were quantified using MTT colorimetric analysis. Findings are presented as percentages relative to control values. Results presented as mean ± SD (n = 6/group). *p < 0.05; **p < 0.01 BMSC: bone marrow mesenchymal stem cell

BMSCs were treated with an appropriate concentration of levofloxacin after 24 hours and had no obvious change in cell number. However, this therapy persisted for two days, and the MTT assay was utilized to evaluate the viability of BMSCs, showing that with the increase of drug concentration, the number of corresponding BMSCs reduced. The MTT assay results revealed a concentration-dependent reduction in cell viability following 48 hours of levofloxacin treatment. Cell viability decreased to 95.17%, 92.90%, 88.69%, 84.67%, and 80.83% at 14 μM, 28 μM, 56 μM, 112 μM, and 224 μM concentrations, respectively (Figure 1).

Annexin V-FITC/PI assay

**Figure 2:**
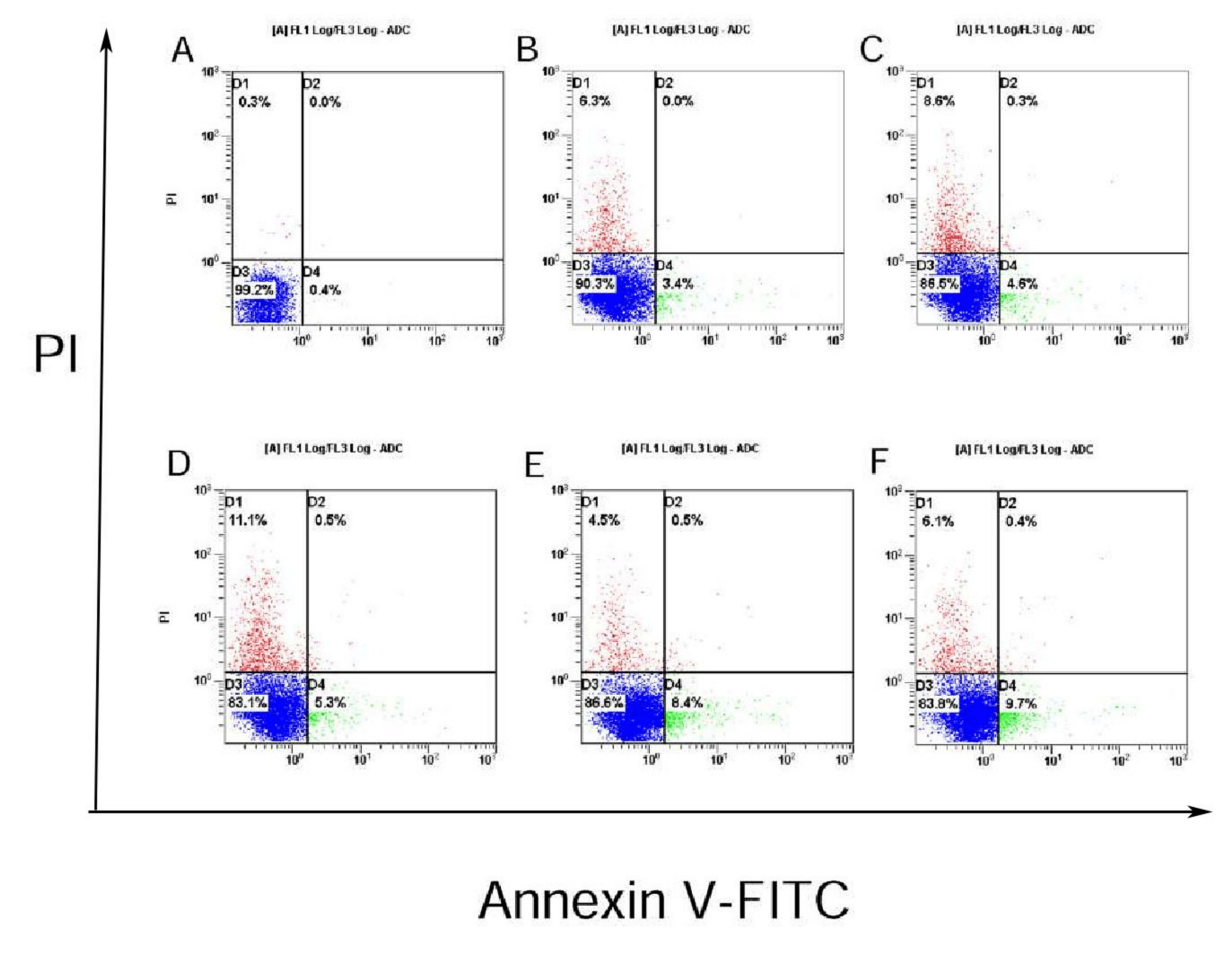
Apoptosis analysis in BMSCs following levofloxacin treatment using Annexin V-FITC/PI staining A: Controlled BMSc; B-F: BMSCs exposed to levofloxacin for 48 hours at doses of 14 μM, 28 μM, 56 μM, 112 μM, and 224 μM, respectively (n = 5/group). The proportion of cells in each form was computed utilizing the Cell Quest Pro software program (mean values provided; the investigation was repeated thrice). The quadrants in each plot are labeled as follows: upper right (UR): necrotic cells, lower right (LR): early apoptotic cells, lower left (LL): viable cells. PI: propidium iodide; FITC: fluorescein isothiocyanate

According to Annexin V-FITC/PI staining, higher concentrations of levofloxacin were associated with a greater proportion of apoptotic cells. After 48 hours of treatment with 14 μM to 224 μM levofloxacin, the proportion of cells that underwent apoptosis increased to 3.4%, 4.5%, 5.3%, 8.4%, and 9.7%, respectively, indicating that levofloxacin promotes apoptosis in BMSCs (Figure 2).

RT-PCR analysis

**Figure 3:**
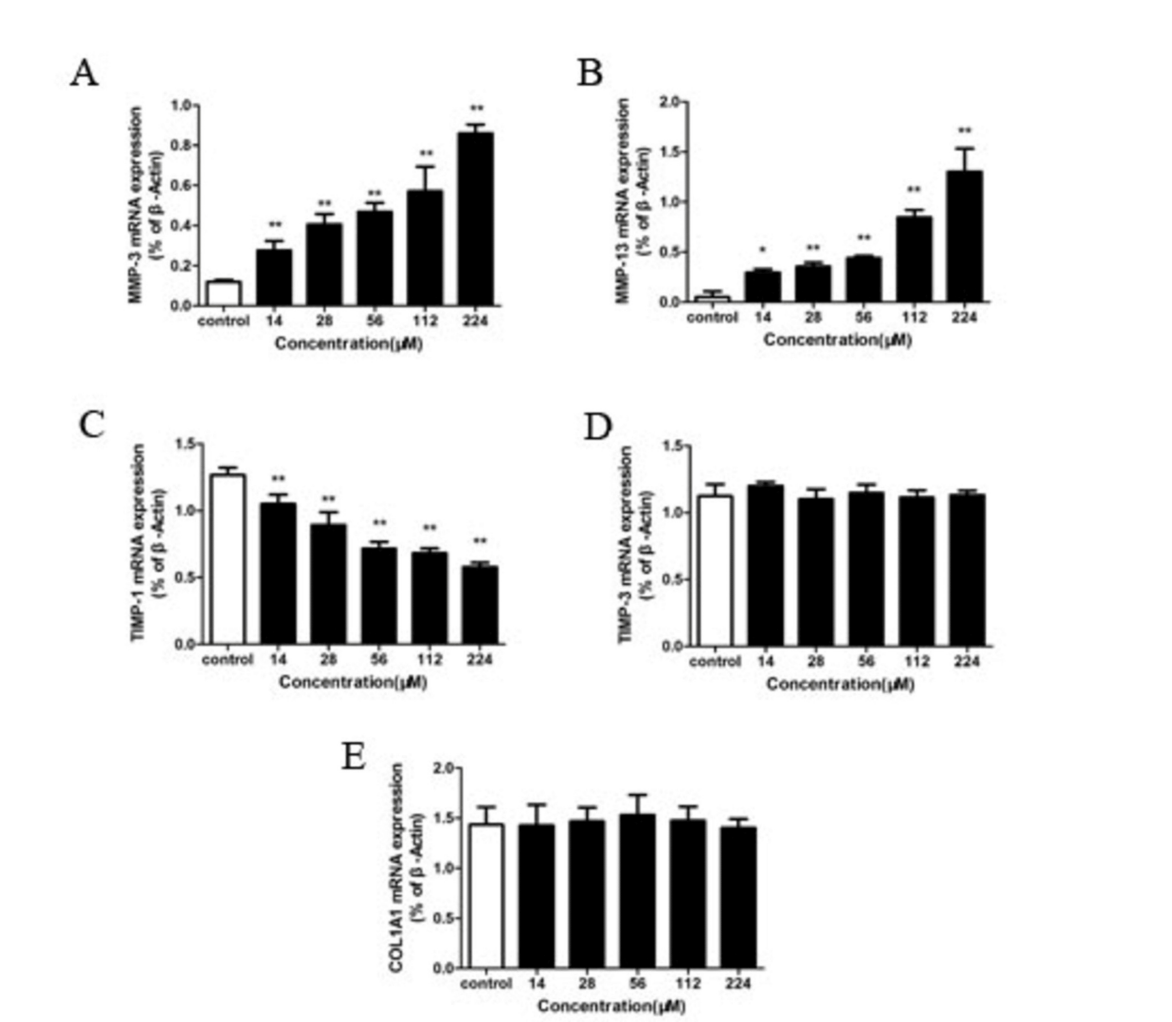
Levofloxacin short-duration (24 hours) impact on BMSCs targeted gene expression A-E: RT-PCR mRNA expression of MMP-3, MMP-13, TIMP-1, TIMP-3, and COL1A1 after Levofloxacin therapy at various dosages. The findings were expressed as a ratio relative to the control (β-Actin). Every strip illustrates the mean ± SD (n = 6/group). *p < 0.05; **p < 0.01 BMSC: bone marrow mesenchymal stem cell; RT-PCR: reverse transcriptase polymerase chain reaction; mRNA: messenger RNA; MMP: matrix metalloproteinase; TIMP: tissue inhibitor of metalloproteinase; Col1A1: collagen type I alpha 1

To explore specific gene expression, RT-PCR analysis was performed on BMSCs handled with levofloxacin at various concentrations of 14 μM, 28 μM, 56 μM, 112 μM, and 224 μM during a period of 24 hours. According to the data shown in Figure 3, it is evident that the mRNA expression of MMP-13 along with MMP-3 was considerably surged by levofloxacin in a way that was dependent on the concentration (p < 0.05). Conversely, the mRNA levels of TIMP-1 were notably reduced (p < 0.01) under similar conditions. However, the expression levels of Col1A1 and TIMP-3 remained stable.

Immunocytochemical analysis

**Figure 4:**
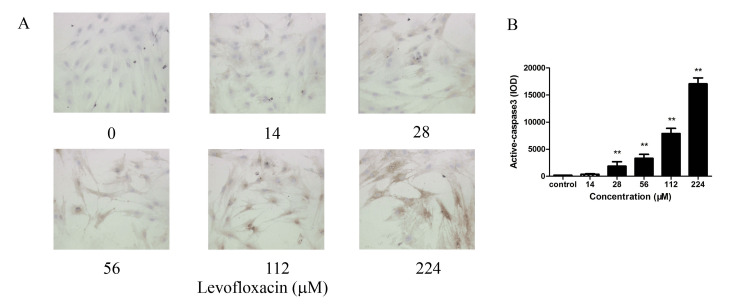
Immunocytochemical detection of active caspase-3 secretion in BMSCs following 48-hour treatment with levofloxacin A: Morphological ultrations in BMSCs following active caspase-3 expression; B: Staining intensity was quantified by assessing the IOD using the Image Pro Plus 6.0 software in 10 various fields in each sample. Results illustrated mean ± SD (n = 10/group). *p < 0.05; **p < 0.01 BMSC: bone marrow mesenchymal stem cell; IOD: integrated optical density

We detect the expression of caspase 3 by immunocytochemical analysis in each group. The results are shown in Figure 4, in a certain concentration range (14 μM to 224 μM); with increasing the concentration of levofloxacin, the expression of apoptosis markers caspase-3 increased significantly (p < 0.01).

## Discussion

The use of quinolones in therapeutic settings is common, and some of their side effects have been reported previously. Some studies have shown that quinolones will affect the growth and differentiation of the osteoblasts [14]. Apoptosis and disruption of the ECM are key factors in the pathogenesis of quinolone-induced tendon rupture [13,15]. Previous studies have shown that treatment with ciprofloxacin at concentrations greater than 300 μM does not result in a significant reduction in human tendon cell viability [16]. Previous studies have demonstrated significant inhibition of osteoblast function at ciprofloxacin concentrations of 40 μg/ml [17,18]. Similarly, levofloxacin exhibits inhibitory effects on human lymphocytes at 20 μg/ml, with complete suppression of cell growth observed at concentrations between 80 μg/ml and 160 μg/ml. Additionally, some studies have reported significant upregulation of the apoptotic marker caspase-3 at levofloxacin concentrations of 30 μg/ml, with notable increases observed even at lower concentrations (20 μg/ml) [19]. Therefore, we believe that levofloxacin can induce apoptosis in BMSCs.

Fracture healing is a complex biological process. Previous studies have demonstrated that BMSCs respond to injury-site microenvironmental signals by migrating to the trauma location. At the injury site, these cells undergo osteogenic differentiation into osteoblasts, utilizing their differentiation capacity and regenerative properties to facilitate healing [20]. The healing process depends critically on both ECM collagen synthesis and the regulated activity of matrix-degrading enzymes. Type I collagen serves as a fundamental component in this process, supporting osteoblast phenotype maturation and calcium nodule formation. Additionally, it plays an essential role in promoting mesenchymal stem cell differentiation into osteoblasts and enhancing osteoblast adhesion [21].

During the process of fracture healing, both type I and type II collagen play important roles. Our study revealed that BMSCs express MMPs and TIMPs during fracture repair and may participate in matrix remodeling. Previous research has demonstrated that MMP-3 and MMP-13 can degrade multiple collagen types (I, II, III, and IV) [22,23]. By degrading ECM components, MMPs facilitate angiogenesis, making them crucial mediators of bone formation and vascularization [24]. TIMPs function as specific inhibitors of MMPs, exerting effects opposite to those of MMPs. Following fracture, MMPs and TIMPs expressed by BMSCs are involved in fracture repair. Our findings indicate that elevated levels of MMPs, coupled with altered TIMP expression, correspond to enhanced matrix degradation. An imbalance in MMPs and TIMP expression can reduce collagen type I expression, further contributing to ECM destruction. These findings suggest that levofloxacin treatment may increase the risk of delayed or impaired fracture healing in patients. BMSCs, derived from bone marrow stroma, possess both self-renewal capacity and multi-lineage differentiation potential. These properties make them ideal for cell therapy and tissue engineering applications [25]. Clinical studies have demonstrated BMSCs' effectiveness in promoting tissue regeneration and repair in various conditions, including arthritis and myocardial infarction [26]. Within the bone marrow microenvironment, these cells serve as essential components, contributing to bone tissue regeneration by providing osteoblast precursors. Furthermore, BMSCs regulate hematopoietic stem and progenitor cell (HSPC) development through both direct cellular interactions and paracrine signaling [27]. Our findings demonstrate levofloxacin's cytotoxic effects on BMSCs, leading to apoptosis. These effects may contribute to delayed fracture healing and impaired tissue regeneration. Moreover, levofloxacin's impact on BMSCs could potentially compromise both transplantation efficacy and normal hematopoietic function.

Following a standard oral dose of levofloxacin (500 mg) in humans, plasma concentrations peak between 13.3 μM and 15.5 μM (4.7-5.7 μg/ml), with an area under the curve (AUC) ranging from 61 μM to 168.8 μM (45-61 μg/ml). Therefore, the concentration levels of levofloxacin used in this study are comparable to those potentially reaching the fracture site. The effects observed at these doses may offer valuable insights, at least partially, for informing clinicians.

This study has several limitations, as our investigation was limited to in vitro conditions, which may not fully reflect the complex physiological in vivo. The study focused exclusively on levofloxacin, and the effects of other fluoroquinolones on BMSCs remain unexplored. Additionally, while we observed cellular changes, the complete molecular mechanisms underlying levofloxacin-induced cytotoxicity require further investigation.

## Conclusions

The results of our study provide evidence that levofloxacin causes apoptosis in rat BMSCs. Another important pathogenic mechanism that is involved in levofloxacin-induced cytotoxicity in BMSCs in vitro is programmed cell death. In addition, levofloxacin has the potential to upset the equilibrium between MMPs and TIMPs, which results in an increased breakdown of the ECM in BMSCs. According to the findings of our research, levofloxacin is cytotoxic to BMSCs in vitro, which indicates that it may have consequences in vivo.
